# Reversible Adrenal Insufficiency in Three Patients With Post–Roux-en-Y Gastric Bypass Noninsulinoma Pancreatogenous Hypoglycemia Syndrome

**DOI:** 10.1177/2324709614526992

**Published:** 2014-03-13

**Authors:** Shelly Mathur, Jasmine Boparai, Sanjay N. Mediwala, Jose M. Garcia, Glenn R. Cunningham, Marco Marcelli, Madhuri M. Vasudevan

**Affiliations:** 1 Baylor College of Medicine, Houston, TX, USA; 2 Michael E. DeBakey VA Medical Center, Houston, TX, USA; 3 University of Texas Medical Branch, Galveston, TX, USA

**Keywords:** adrenal insufficiency, noninsulinoma pancreatogenous hypoglycemia syndrome, Roux-en-Y gastric bypass

## Abstract

*Objective*. Noninsulinoma pancreatogenous hypoglycemia syndrome (NIPHS) is a disorder of endogenous hyperinsulinemia that is clinically distinguishable from insulinoma, with a greater preponderance after Roux-en-Y gastric bypass (RYBG). Hyperinsulinemic hypoglycemia can predispose to attenuation of counterregulatory hormone responses to hypoglycemia, and consequent suppression of the hypothalamic–pituitary–adrenal (HPA) axis. This case series describes 3 individuals who were diagnosed with adrenal insufficiency (AI) after undergoing RYGB, complicated by NIPHS. *Methods*. A retrospective chart review was performed for each individual. Chart review applied particular attention to the onset of hyperinsulinemic hypoglycemia following bariatric surgery and the dynamic testing leading to the diagnoses of NIPHS and AI. *Results*. In each case, reactive hypoglycemia ensued within months to years after RYGB. Cosyntropin stimulation testing confirmed the diagnosis of AI. Hydrocortisone therapy reduced the frequency and severity of hypoglycemia and was continued until successful medical and/or surgical management of hyperinsulinism occurred. Follow-up testing of the HPA axis demonstrated resolution of AI. In all cases, hydrocortisone therapy was finally discontinued without incident. *Conclusion*. We speculate that transient AI is a potential complication in patients who experience recurrent hyperinsulinemic hypoglycemia after RYGB. The putative mechanism for this observation may be attenuation of the HPA axis after prolonged exposure to severe, recurrent hypoglycemia. We conclude that biochemical screening for AI should be considered in individuals who develop post-RYGB hyperinsulinemic hypoglycemia. If AI is diagnosed, supportive treatment should be maintained until hyperinsulinemic hypoglycemia has been managed effectively.

## Introduction

Disorders of endogenous hyperinsulinemia include insulinoma, persistent hyperinsulinemic hypoglycemia of infancy (PHHI), and noninsulinoma pancreatogenous hypoglycemia syndrome (NIPHS). Risk factors and mechanisms for the spontaneous development of NIPHS remain unclear. However, NIPHS appears to develop with increased frequency among patients who have undergone Roux-en-Y gastric bypass (RYGB).^1,2^ Although pancreatic histopathology of patients with NIPHS resembles pancreatic specimens from infants with PHHI,^3^ the genetic mutations observed in PHHI are not observed in patients with NIPHS.^4^ NIPHS can be distinguished from insulinoma due to the presence of neuroglycopenia in the postprandial state, euglycemia during prolonged fasts, no localization on imaging studies, and presence of an increased insulin response in veins that drain one or more regions of the pancreas during selective arterial calcium stimulation testing.^5^ Proposed etiologies for NIPHS include the development of increased incretin levels, which may contribute to augmented beta-cell function or growth,^6^ and consequent recurrent severe hypoglycemia.

Attenuated counterregulatory hormonal responses to severe hypoglycemia occur in patients with insulinoma,^7-11^ in infants with PHHI,^12,13^ and in diabetic patients treated with excess exogenous insulin.^14,15^ Insulinoma resection mitigates hyperinsulinemic hypoglycemic episodes and is associated with restoration of physiologic counterregulatory hormone response to hypoglycemia.^7-11^ We report 3 individuals who underwent RYGB complicated by NIPHS and subsequently developed adrenal insufficiency (AI). Supportive hydrocortisone therapy reduced severity and frequency of hypoglycemia and was continued until effective management of hyperinsulinism permitted recovery from pathologic hypoglycemia. Thereafter, endogenous glucocorticoid function recovered in all cases.

## Methods

This retrospective case series was authorized by the institutional review board of Baylor College of Medicine and the Research and Development Committee at the Michael E. DeBakey Veterans Affairs Medical Center. Chart review applied particular attention to the onset of hyperinsulinemic hypoglycemia following bariatric surgery and the dynamic testing leading to the diagnoses of NIPHS and AI.

### Case 1

A 53-year-old woman with morbid obesity underwent RYGB in March 2008. Two years later, she reported frequent postprandial hypoglycemic episodes. The patient underwent 75-g oral glucose tolerance test (OGTT). Ninety minutes after glucose load, plasma glucose decreased to 31 mg/dL, with concurrent serum insulin 41 µIU/mL, C-peptide 5.3 ng/mL, serum cortisol 10.5 µg/dL, and negative sulfonylurea screen (Table 1). The patient’s clinical presentation, in the context of inappropriately low cortisol (<18 µg/dL) response to severe hypoglycemia during OGTT, raised the suspicion for AI, and the patient initiated physiologic hydrocortisone replacement therapy as a precaution. Dynamic testing of the hypothalamic–pituitary–adrenal (HPA) axis with 1 µg cosyntropin stimulation confirmed the AI diagnosis. Anti-adrenal antibodies were negative. Computed tomography of the pancreas revealed no areas of abnormal enhancement or mass lesion. Selective arterial calcium stimulation testing caused increased insulin levels in veins, draining the distal pancreas. α-Glucosidase inhibitor, diazoxide, and somatostatin analogue therapies were initiated and reduced the frequency of hypoglycemic episodes. Despite optimization of medical therapy, the frequency and severity of hypoglycemia necessitated distal pancreatectomy. Pancreatic pathology revealed several foci of islet cell hyperplasia, consistent with a histopathologic diagnosis of nesidioblastosis. Hydrocortisone therapy continued in the perioperative period and was discontinued 2 months after surgery. Follow-up testing with 250 µg cosyntropin stimulation revealed adequate cortisol response. She has remained clinically stable to date on medical and dietary management of reactive hypoglycemia. The patient’s total duration of hydrocortisone therapy was 9 months.

**Table 1.** Oral Glucose Tolerance Test Revealed Reactive Hypoglycemia With Inappropriately High Insulin Level and Inappropriately Low Serum Cortisol^a^.

	NIPHS Diagnosis	AI Diagnosis	AI Resolution
Case 1	75 g OGTT	1 µg CST	250 µg CST
Date	April 2011	June 2011	August 2012
Biochemical test, unit, reference range			
Plasma glucose, mg/dL, 70-100	31 (90 minutes)		
Cortisol, µg/dL, >18^b^	10.5		
Insulin, µIU/mL, 0-24.9	41		
C-Peptide, ng/mL, 1.1-4.4	5.3		
Cosyntropin stimulation test			
Cortisol baseline, µg/dL, >10.8^b^		10.3	6.6
Cortisol 30 minutes poststimulation, µg/dL, >18^b^		17.2	21.4
Cortisol 60 minutes poststimulation, µg/dL, >18^b^		12.7	22.7

Abbreviations: NIPHS, noninsulinoma pancreatogenous hypoglycemia syndrome; AI, adrenal insufficiency; CST, cosyntropin stimulation test; OGTT, oral glucose tolerance test; HPA axis, hypothalamic–pituitary–adrenal axis.

a Patient was diagnosed with adrenal insufficiency based on inappropriate cortisol response to 1 µg cosyntropin stimulation. Definitive medical and surgical intervention resulted in reduced frequency and severity of hypoglycemia. Subsequent testing of the HPA axis revealed intact cortisol response to cosyntropin stimulation.

b Cortisol thresholds based on References 17 and 18.

### Case 2

This man presented at age 37 years with a history of obesity, hyperlipidemia, obstructive sleep apnea, gastroesophageal reflux disease, and lumbar radiculopathy. He underwent RYGB in 2008. One year later, he presented to endocrine clinic with symptomatic hypoglycemia. During a 72-hour fasting test, his blood glucose level decreased to 45 mg/dL after 24 hours. Concurrent labs included serum insulin of 87 µIU/mL, C-peptide of 9.7 ng/dL, cortisol of 14.2 µg/dL, and negative sulfonylurea screen (Table 2). Serum cortisol level <18 µg/dL with severe hypoglycemia raised the suspicion for AI, and the patient initiated physiologic hydrocortisone replacement therapy as a precaution. Shortly thereafter, 1 µg cosyntropin stimulation confirmed the diagnosis of AI (Table 2). Computed tomography of the pancreas revealed no mass lesions. Selective arterial calcium stimulation increased insulin levels in the veins drained by the gastroduodenal and proximal splenic arteries. Somatostatin analogue therapy was initiated, and hypoglycemic symptoms improved. The patient’s hypoglycemic episodes have decreased in severity and frequency. A follow-up 250 µg cosyntropin stimulation test revealed an adequate serum cortisol response (Table 2), and hydrocortisone therapy was discontinued. Patient’s total duration of hydrocortisone therapy was 18 months.

**Table 2.** 72-Hour Fast Revealed Hypoglycemic Hyperinsulinism, With Inappropriately Low Serum Cortisol, Raising Suspicion for Adrenal Insufficiency^a^.

	NIPHS Diagnosis	AI Diagnosis	AI Resolution
Case 2	72-Hour Fast	1 µg CST	250 µg CST
Date	March 2009	June 2009	September 2009
Biochemical test, unit, reference			
72-hour fasting test			
Plasma glucose, mg/dL, 70-100	45		
Cortisol, µg/dL, >18^b^	14.2		
Insulin, µIU/mL, 0-24.9	87		
C-Peptide, ng/mL, 1.1-4.4	9.7		
1 µg cosyntropin stimulation test			
Cortisol baseline, µg/dL, >10.8^b^		7.27	10.8
Cortisol 30 minutes poststimulation, µg/dL, >18^b^		4.9	22.2
Cortisol 60 minutes poststimulation, µg/dL, >18^b^		11.48	ND

Abbreviations: NIPHS, noninsulinoma pancreatogenous hypoglycemia syndrome; AI, adrenal insufficiency; CST, cosyntropin stimulation test; ND, not done.

a Patient was diagnosed with adrenal insufficiency by 1 µg cosyntropin stimulation testing. After medical management of adrenal insufficiency and definitive therapy for hyperinsulinemic hypoglycemia, cosyntropin stimulation demonstrated recovery of the hypothalamic pituitary adrenal axis.

b Cortisol thresholds based on References 17 and 18.

### Case 3

This 39-year-old woman underwent RYGB in 2006. Three years later, she presented with postprandial hypoglycemic episodes. Diagnostic continuous glucose monitoring data revealed pattern of recurrent 1 to 3 hour postprandial interstitial (51 mg/dL and 47 mg/dL) and capillary (48 mg/dL and 59 mg/dL) glucose levels associated with hypoglycemic symptoms over 2 consecutive days. These results promoted dynamic testing with a 72-hour fast, which revealed no plasma glucose levels less than 70 mg/dL. However, due to clinical suspicion of NIPHS, she underwent selective arterial calcium stimulation test, which demonstrated hyperinsulinism localized to the veins draining the superior mesenteric, splenic, and gastroduodenal arteries. Despite undergoing an 80% pancreatectomy, which revealed normal pancreatic tissue, her hypoglycemic symptoms persisted. In January 2009, she was admitted for orthostatic hypotension, at which time she underwent a 250 µg cosyntropin stimulation test that revealed an inadequate cortisol response (Table 3), and treatment with hydrocortisone was started. Her hypoglycemic episodes abated when she was treated with an α-glucosidase inhibitor. After 20 months of hydrocortisone therapy, an early morning serum cortisol measured 14.8 µg/dL (Table 3). This value was interpreted as sufficient level to reflect an intact hypothalamic pituitary adrenal axis,^18^ and hydrocortisone therapy was discontinued. She has remained clinically stable to date.

**Table 3.** Patient Was Diagnosed With Adrenal Insufficiency After Partial Pancreatectomy, in the Context of Persistent Hypoglycemia, Given Inappropriately Low Cortisol Response to 250 µg Cosyntropin Stimulation Testing^a^.

	AI Diagnosis	AI Resolution
Case 3	250 µg CST	AM Cortisol
Date	January 2009	August 2010
Biochemical test, unit, reference		
Cortisol baseline, µg/dL, >10.8^b^	1	14.8
Cortisol 30 minutes poststimulation, µg/dL, >18^b^	9.9	ND
Cortisol 60 minutes poststimulation, µg/dL, >18^b^	13.5	ND

Abbreviations: AI, adrenal insufficiency; CST, cosyntropin stimulation test; ND, not done; RYGB, Roux-en-Y Gastric Bypass;.

a Despite undergoing partial pancreatectomy after RYGB, this patient continued to experience hypoglycemic episdoes. Cosyntropin stimulation confirmed AI. After 20 months of hydrocortisone replacement therapy and optimizing medical management of hyperinsulinemic hypoglycemia, cosyntropin stimulation demonstrated recovery of the hypothalamic–pituitary–adrenal axis.

b Cortisol thresholds based on References 17 and 18.

## Discussion

Two of the 3 patients with hyperinsulinemic hypoglycemia described in this report (Cases 1 and 2) developed post-RYGB AI prior to medical or surgical therapy of hypoglycemia, and the third patient was diagnosed with post-RYGB AI after a distal pancreatectomy. Histopathologic evidence of nesidioblastosis was identified in case 1, but not in case 3; however, the absence of nesidioblastosis does not preclude the diagnosis of NIPHS.^19^ Although the cortisol responses observed during OGTT in case 1 and 72-hour fast in case 2 were considered inadequate and raised the suspicion for AI, the diagnosis of AI was confirmed by cosyntropin stimulation. In cases 1 and 2, physiologic hydrocortisone preceded medical and/or surgical therapies aimed at reducing endogenous hyperinsulinism, which ultimately permitted recovery from pathologic hypoglycemia. In case 3, however, persistence of hypoglycemia and orthostatic hypotension after pancreatectomy led to the diagnosis of AI. Although pancreatectomy may have eliminated the source of hyperinsulinism, the patient required hydrocortisone support until objective evidence of adrenal function recovery. In all 3 cases, supportive hydrocortisone therapy reduced the severity and frequency of hypoglycemia. Duration of physiologic steroid replacement for AI varied in each case. Follow-up dynamic testing with cosyntropin stimulation demonstrated recovery of the hypothalamic pituitary adrenal axis, mitigating the need for continued treatment.

Idiopathic NIPHS is considered a rare condition of unclear etiology. However, there appears to be increased risk to develop acquired NIPHS following RYGB. Bariatric surgery is associated with significant weight loss, improves glycemic control, and may decrease cardiovascular mortality as compared to medical therapy.^20^ NIPHS is a known complication of RYGB and can occur months to years after RYGB.^1,2^ This condition is diagnosed in approximately 0.21% of individuals who have undergone gastric bypass surgery, and it is more common in women.^21^ Medical therapy and/or gradient-guided surgical resection of the involved pancreas can attenuate the severity and frequency of hyperinsulinemic hypoglycemia in NIPHS.^5^

Historically, individuals at greatest risk for recurrent hyperinsulinemic hypoglycemia were patients with endogenous hyperinsulinism due to insulinoma or PHHI or exogenous hyperinsulinism in diabetic patients receiving excessive insulin therapy.^7-15^ Diabetic patients treated with intensive insulin therapy have a 3-fold increased risk of severe, recurrent hypoglycemia and demonstrate defective counterregulatory responses affecting cortisol, glucagon, GH, and catecholamines.^15^ In type 1 and advanced type 2 diabetic patients, iatrogenic hypoglycemia predisposes to hypoglycemia-associated autonomic failure, which is thought to arise from defective glucose counterregulation and reduced sympathoadrenal responses to subsequent hypoglycemia. The mechanism by which hypoglycemia shifts the glycemic thresholds for sympathoadrenal activation is unknown. What has been observed is that meticulous avoidance of hypoglycemia can reverse the pathophysiologic mechanisms responsible for hypoglycemia associated autonomic failure.^16^

Similarly, in a patient with chronic insulinoma-induced recurrent hypoglycemia, counterregulatory hormone responses to hypoglycemia were elicited at a lower glycemic threshold, and the absolute increase in counterregulatory hormone (cortisol, epinephrine, and norepinephrine) levels was less than that observed in the same patient after insulinoma resection.^7^ Insulinoma resection and restoration of euglycemia raises the glycemic threshold for counterregulatory hormone response to hypoglycemia and increases the amplitude of that response.^7-10^

Putative mechanisms responsible for adaptive responses to severe recurrent hypoglycemia in this subset of patients include antecedent hypoglycemia-induced suppression of endogenous counterregulatory hormone responses to subsequent hypoglycemia and recurrent exposure of the HPA axis to hypoglycemia-induced hypercortisolism, which directly suppresses subsequent responses to severe hypoglycemia. Studies of type 1 and type 2 diabetics with hypoglycemia-associated autonomic failure identify various mechanisms for pathophysiologic responses to recurrent hypoglycemia, including loss of glucagon response, reduced sympathetic neural activation to subsequent hypoglycemia, and reduced sympathoadrenal responses to subsequent hypoglycemia.^16,22^

In normal individuals who receive an exogenous bolus of insulin, the ensuing acute hypoglycemic response is associated with a significant rise in systemic concentrations of plasma corticotrophin releasing hormone (CRH),^23^ suggesting that CRH plays a role as a physiological mediator of adrenocorticotropic hormone (ACTH) secretion, when induced by hypoglycemic stress. Animal studies have confirmed a role for the CRH surge in counterregulatory hormone response to hypoglycemia, demonstrating that acute intracerebroventricular administration of CRH was associated with a concomitant activation of the HPA axis, while chronic administration resulted in an attenuated release of ACTH and cortisol,^24^ possibly via downregulation of the CRH receptor.^25^ Hypercortisolism itself, which is a response to acute hypoglycemia, can reduce autonomic neuroendocrine and neurogenic responses to subsequent hypoglycemic episodes.^26^ Antecedent hypoglycemia has been demonstrated in healthy controls to blunt glucagon, ACTH, and hepatic glucose production during subsequent hypoglycemia, suggesting that counterregulatory hypercortisolemic response to hypoglycemia may itself play a causative role in the subsequent inadequate cortisol responses to hypoglycemia.^27^

This case series seeks to bring attention to a unique and compelling clinical observation that persistent endogenous hyperinsulinemic hypoglycemia after RYGB may contribute to the development of AI. The clinical hypothesis for the etiology behind this observation is that recurrent severe hypoglycemia and hypoglycemia-induced hypercortisolemic insults to the HPA axis results in downregulation of counterregulatory hormone responses, including glucocorticoid levels. In such patients, insulin tolerance testing may not be test of choice, due to an anticipated attenuation in physiologic response to acute insulin-induced hypoglycemia. Therefore, cosyntropin stimulation testing may be considered the preferred dynamic test. The choice between 1 µg and 250 µg cosyntropin stimulation remains challenging, given the operator variability that can occur with 1 µg dilution of cosyntropin. From a physiological perspective, it would appear that 1 µg cosyntropin stimulation test may be considered preferable, when the clinical suspicion supports a diagnosis of central AI.^28^

Limitations to this case series include the retrospective nature of the observation and the variability in the approach to diagnosis of reactive hypoglycemia and AI. Another limitation to this case series is the lack of additional biochemical evidence to support the clinical suspicion of central AI, such as adrencorticotropic hormone or growth hormone levels. Although the suggested hypothesis regarding the pathophysiological basis for development of AI and the reasons for recovery from AI in this series are grounded in animal and human studies of HPA physiological response to acute and chronic hypoglycemia and hypercortisolemic response to hypoglycemia, clinical studies within this particular subset of patients regarding the association of AI and post-RYBG have yet to be performed. Future research in post-RYGB patients in a controlled and prospective manner is warranted to validate the observations of this case series.

To our knowledge, this is the first report suggesting that AI is a potential complication associated with NIPHS after RYGB. Physiologic hydrocortisone replacement therapy reduces the severity and frequency of hypoglycemia. Ultimately, medical and/or surgical amelioration of endogenous hyperinsulinemia-induced hypoglycemia appears therapeutically necessary. Thus, in cases of recurrent, severe hyperinsulinemic hypoglycemia after RYGB, AI may be suspected, and physiologic glucocorticoid replacement can be supportive until definitive management of hyperinsulinemic hypoglycemia has been achieved.
